# The Impact of Atorvastatin Treatment on the Distribution of Low-Density Lipoprotein Subfractions and the Level of Vitamin D in Patients After Acute Myocardial Infarction: Preliminary Findings

**DOI:** 10.3390/ijms252011264

**Published:** 2024-10-19

**Authors:** Grażyna Sygitowicz, Dariusz Sitkiewicz, Karol Wrzosek, Mirosław Dłuźniewski

**Affiliations:** 1 Department of Medical Laboratory, Faculty of Pharmacy, Medical University of Warsaw, 1 Banacha Str., 02-097 Warsaw, Poland; dariusz.sitkiewicz@gmail.com; 2 Department of Heart Diseases, Postgraduate Medical School, Masovian Brodnowski Hospital, 8 Kondratowicza Str., 03-242 Warsaw, Poland; wrzosekk@poczta.onet.pl (K.W.); dluzniewski@ptkardio.pl (M.D.)

**Keywords:** atorvastatin, lipoprotein subfractions, triglyceride-rich lipoproteins, lipid profile, 25(OH)D, acute myocardial infarction

## Abstract

Clinical trial results indicate that statin therapy aimed at normalising the lipid profile can prevent and reduce the risk of cardiovascular events. Both LDL and HDL consist of several subfractions, with only the smallest and densest subfractions being the most atherogenic. We examine the effect of Atorvastatin treatment not only on basic lipid profile parameters but also atherogenic lipoprotein subfractions and 25(OH)D levels in patients after the first acute myocardial infarction. The study population had not previously received lipid-lowering medications. Serum 25(OH)D concentration was determined by direct competitive immunochemiluminescent assays. Lipoprotein subfractions, including VLDL, IDL-C, IDL-B, and IDL-A, as well as LDL1, LDL2 (large LDL), and LDL3-7 (sdLDL), were measured in serum (Lipoprint^®^ system). Almost all patients had 25(OH)D deficiency. Atorvastatin primarily reduced strongly atherogenic sdLDL and decreased the less atherogenic large LDL subfractions. A statistically significant reduction in VLDL cholesterol and IDL fractions was also observed. Analysing LDL subfractions provides a more detailed insight into lipid metabolism and enables the identification of patients with a more atherogenic phenotype. LDL subfractions may thus become not only more accurate prognostic biomarkers but also targets for lipid-lowering therapy. Vitamin D deficiency is associated with atherogenic dyslipidaemia, particularly high levels of sdLDL.

## 1. Introduction

Cardiovascular diseases are among the leading causes of mortality in many countries [1,2]. Various factors, especially obesity, an inactive lifestyle, stress, and diseases such as diabetes and dyslipidaemia, increase the risk of cardiovascular complications. Elevated low-density lipoprotein (LDL) levels and reduced high-density lipoprotein (HDL) levels are key risk factors for cardiovascular diseases [3]. Both LDL and HDL consist of several subfractions, with the smallest and densest subfractions being the most atherogenic. Electrophoretic separation of LDL fractions reveals seven subfractions, ranging from the largest to the smallest. LDL1 and LDL2 are large subfractions, while LDL3-7 are small and dense. A higher content of small, dense LDLs (sdLDLs) is associated with an increased risk of cardiovascular diseases [4]. Small, dense LDL particles are formed when the triglycerides contained in LDLs undergo lipolysis due to the activity of hepatic lipase or other lipases [5,6]. Due to their smaller size or greater propensity for oxidation, the cholesterol contained in sdLDLs may be more proatherogenic than that in other lipoproteins containing apoB [7,8]. Additionally, sdLDL particles exhibit a low affinity for LDL receptors, which leads to a reduced rate of their catabolism and longer circulation time. Clinical trials suggest that statin therapy, which lowers LDL cholesterol, may prevent and reduce the risk of cardiovascular events. Consequently, LDL cholesterol-lowering therapies are recommended by both European [9,10] and American [11] guidelines for cardiovascular disease prevention.

Many studies have shown a relationship between vitamin D deficiency and dyslipidaemia [12,13]. Important findings on the impact of vitamin D on the lipid profile were reported by Ponda et al. [14]. In their study [14], serum 25(OH)D concentrations and lipid profile parameters were analysed in a large group of patients (108,711 people). The results indicated that higher serum concentrations of 25(OH)D correlated with lower levels of total cholesterol, LDL fractions, and triglycerides, as well as higher levels of HDL cholesterol in both men and women [14].

Therefore, the question of how vitamin D affects cholesterol levels is important. However, the mechanisms remain unclear [15]. Vitamin D and cholesterol share similarities in their biosynthesis: cholecalciferol is synthesised in the skin from 7-dehydrocholesterol (7DHC) under UV-B radiation, while 7DHC is also converted to cholesterol by the enzyme 7-dehydrocholesterol reductase (DHCR7) [16]. Zou and Porter [17] demonstrated in vitro that elevated cholecalciferol levels rapidly decrease DHCR7 activity, resulting in a reduction in cholesterol concentration.

One of the primary functions of vitamin D is regulating calcium metabolism, which may influence cholesterol production through various mechanisms. By increasing intestinal calcium absorption, vitamin D may modulate microsomal triglyceride transfer protein (MTP), thereby reducing triglyceride synthesis and secretion [18].

Higher intestinal calcium levels also decrease fatty acid absorption by forming insoluble calcium-fat complexes. Moreover, calcium may promote the conversion of cholesterol to bile acids, thereby lowering cholesterol levels. 25(OH)D regulates parathyroid hormone (PTH) levels, and hyperparathyroidism has been associated with elevated triglyceride levels [19]. Therefore, 25-OH-vitamin D, through PTH regulation, may influence triglyceride levels [19].

Additionally, vitamin D deficiency may affect pancreatic beta cell function and insulin resistance, contributing to altered lipoprotein metabolism, which can result in increased triglyceride levels and decreased HDL cholesterol levels [6]. Low vitamin D levels are also linked to a pro-inflammatory state [20]. Furthermore, calcitriol (1,25(OH)_2_D), the active form of vitamin D, appears to inhibit cholesterol uptake by macrophages, thereby reducing foam cell formation, which is involved in atherogenesis [21].

Statins are among the most commonly prescribed drugs, and many studies have confirmed their efficacy in reducing cardiovascular morbidity and mortality in both primary and secondary prevention [22,23,24]. It is widely accepted that the beneficial effects of statins are not solely due to their inhibition of cholesterol biosynthesis and subsequent reduction in blood cholesterol levels. The broad use of statins is likely related to their multiple pleiotropic effects [23,24]. One such effect may be their influence on vitamin D metabolism. Recent studies have shown that statins increase 25(OH)D concentrations in patients with familial hypercholesterolaemia and ischaemic heart disease [25,26]. Furthermore, Atorvastatin has been found to elevate 25(OH)D levels in patients with polycystic ovary syndrome [27].

The aim of our study was to assess the impact of Atorvastatin treatment on the concentration of atherogenic lipoprotein subfractions and vitamin D levels in patients who experienced their first acute myocardial infarction. Circulating 25(OH)D is the most reliable indicator of vitamin D status in humans, reflecting dietary intake, supplementation, or skin synthesis of vitamin D [28]. Studying the selected population allowed us to determine the vitamin D status of patients immediately after an acute coronary event.

## 2. Results

**Figure 1:**
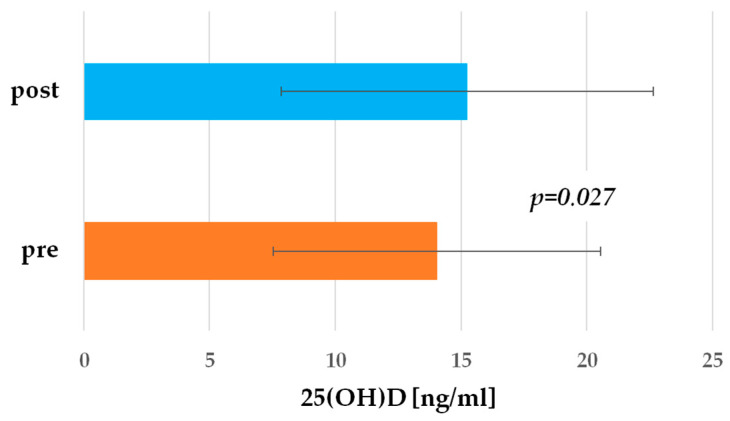
Vitamin D concentrations pre- and post-Atorvastatin treatment. The results of vitamin D concentrations for all patients are presented as mean ± standard deviation.

The mean concentration of 25-hydroxy vitamin D in the study population was 14.19 ± 6.37 ng/mL, with a minimum of 6.1 ng/mL and a maximum of 34.1 ng/mL (Figure 1). In over 82% of cases, 25(OH)D concentrations below 20 ng/mL were found, which indicates significant vitamin D deficiency in almost all patients with acute myocardial infarction.

After 4 weeks of Atorvastatin treatment, 80% of patients still had 25(OH)D levels below 20 ng/mL. The mean 25(OH)D level was 15.52 ± 7.26 ng/mL for the entire study group, indicating an increase in 25(OH)D levels of 8.57% (*p* = 0.027) after Atorvastatin treatment (Figure 1).

The impact of Atorvastatin on the standard lipid profile is presented in Table 1. The levels of all the parameters studied—total cholesterol, HDL-C, non-HDL-C, LDL-C, and triglycerides—were statistically significantly lower after Atorvastatin treatment. Total cholesterol levels decreased by 63.24 ± 41.94 mg/dL, which is a reduction of 32.36%. Similarly, LDL-C concentration decreased by 48.00 ± 31.87 mg/dL, or 41.29%. A statistically significant reduction in HDL-C concentration (by 14.28%) and non-HDL cholesterol (by 39.44%) was also observed. The concentration of triglycerides decreased slightly (by 17.42%), though this reduction was statistically insignificant (Table 1).

**Figure 2:**
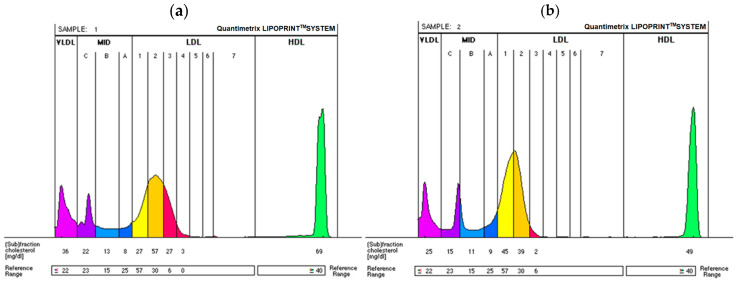
Changes in LDL subfractions pre- and post-Atorvastatin treatment (model lipid electrophoresis for one representative patient). (**a**) Pre-intervention: predominantly phenotype B (not indicative of type A—the presence of small, dense LDL), with atherogenic LDL3-7 subfractions (in red) and large, less atherogenic subfractions LDL1-2 (in yellow). (**b**) Post-intervention: LDL phenotype A, with marked reduction in atherogenic LDL subfractions (mainly large LDL), LDL3-7 (in red), and large, less atherogenic subfractions LDL1-2 (in yellow).

In order to obtain a detailed analysis of the effect of Atorvastatin on the concentrations of individual serum lipoproteins, electrophoretic separation on a polyacrylamide gel (PAG) was performed using the Quantimetrix LipoPrint System. This system will be described in great detail in the Section 4.4 Lipoprotein Subfractions. Lipoprotein analysis was conducted simultaneously during a single electrophoretic separation. The Quantimetrix LipoPrint System separates and quantifies all lipoprotein subfractions (up to 12 fractions), including the large, less atherogenic LDL1 and LDL2 and the small, dense, highly atherogenic LDL3-LDL7. The test also measures the level of VLDL and IDL cholesterol fractions, dividing them into IDL-C, IDL-B, and IDL-A subfractions (Figure 2).

**Figure 3:**
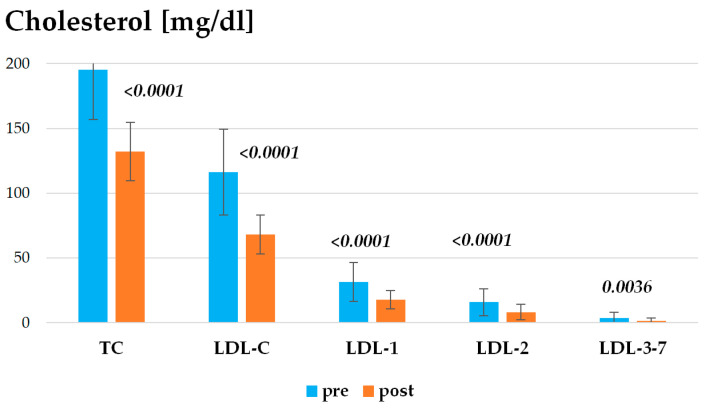
Atherogenic lipoprotein concentrations pre- and post-Atorvastatin treatment. The results of atherogenic lipoprotein concentrations for all patients are presented as mean ± standard deviation.

The detailed results of the electrophoretic studies are also presented in Figure 3. Atorvastatin treatment resulted in a reduction in less atherogenic, large LDL fractions (LDL1 and LDL2), as well as, most importantly, a decrease in the strongly atherogenic sdLDL (LDL3-LDL7).

Although Atorvastatin treatment led to only a slight decrease in triglyceride levels (*p* = 0.1168), our attention was drawn to its effect on triglyceride-rich lipoprotein fractions. Before starting the treatment, elevated (above the reference range) levels of VLDL cholesterol, IDL-C, and IDL-B were observed in the studied patients (Table 2).

After 1 month of Atorvastatin treatment, a statistically significant reduction in the concentration of VLDL cholesterol fraction and all tested IDL fractions was demonstrated. Of particular note is the fact that all triglyceride-rich lipoproteins achieved levels within the reference range following Atorvastatin treatment.

## 3. Discussion

The presented results indicate that patients with acute myocardial infarction (AMI) (including STEMI or NSTEMI) have low 25(OH)D levels, with over 80% of patients showing vitamin D deficiency. Numerous studies have investigated the relationship between vitamin D deficiency and coronary artery disease. The results to date indicate that patients with low vitamin D levels have an increased risk of major adverse coronary events (MACE) [29,30]. Lee et al. [31] studied the prevalence of vitamin D deficiency among patients with AMI and reported that 75% of patients with AMI had vitamin D deficiency, with 21% having insufficient vitamin D levels. This suggests that vitamin D deficiency is present in almost all individuals with AMI. These findings clearly confirm that low 25(OH)D levels are associated with a higher risk of myocardial infarction, even after the normalisation of lipid profile parameters, which are closely linked to coronary artery disease.

Our study shows that Atorvastatin is capable of activating vitamin D synthesis. Yavuz et al. [25] and Ertugrul et al. [26] observed a large increase in 25(OH)D concentration after treatment with Rosuvastatin (to 12.6 ng/mL and to 23.4 ng/mL, respectively). In these studies, patients with hyperlipidaemia were included, and the statin treatment was of a primary prevention nature. However, in our specific group of post-myocardial infarction patients, in whom Atorvastatin treatment was part of secondary prevention, we observed only a relatively small increase in 25(OH)D concentration (1.33 ng/mL).

The presented data confirm the thesis that vitamin D deficiency is associated with atherogenic dyslipidaemia. In our studied group of patients with vitamin D deficiency, we observed the presence of small, dense LDL (atherogenic subfractions LDL3-7) and large, less atherogenic subfractions (LDL1-2). We also examined potential relationships between vitamin D levels and the concentrations of lipoprotein fractions and subfractions, both before and after Atorvastatin treatment. However, we did not find any significant correlations between 25(OH)D concentrations and the studied lipoprotein subfractions in patients both immediately after myocardial infarction and post-Atorvastatin treatment. We did, however, observe a statistically significant correlation between 25(OH)D concentration and non-HDL cholesterol concentration (Rs = −0.342; *p* < 0.05) in patients who experienced myocardial infarction prior to Atorvastatin treatment. Notably, this relationship became insignificant (*p* > 0.05) following treatment. We did not find any correlation after Atorvastatin treatment, which may indicate that even a relatively small increase in vitamin D concentration affects the metabolism of lipoproteins containing apo B. Since Atorvastatin predominantly targets apo-B-rich lipoproteins, the vitamin D/non-HDL cholesterol correlation disappeared after treatment (even though it lasted only 4 weeks). Nevertheless, the absence of other significant correlations may be due to the small sample size and the relatively narrow range of 25(OH)D concentrations both before and after Atorvastatin therapy. This lack of correlation may indicate that the connection between vitamin D levels and lipoprotein metabolism is more complex than initially thought.

In these patients, we also observed elevated concentrations of triglyceride-rich lipoproteins, specifically VLDL and IDL. The most recent Polish recommendations on the laboratory diagnostics of lipid disorders (2024) [32] highlight the importance of these lipoproteins in assessing residual cardiovascular risk. Circulating and tissue lipoprotein lipases hydrolyse the triglycerides contained in VLDL, converting them into VLDL/IDL remnants. These remnants have an increased cholesterol content and are much smaller in size, which contributes to their greater penetration into blood vessels and increased atherogenicity [33,34].

Our findings indicate that Atorvastatin, while having only a modest effect on reducing overall triglyceride levels, significantly modifies the content of triglyceride-rich lipoproteins. In addition to reducing LDL cholesterol concentration, Atorvastatin also improves the profile of LDL subfractions. One significant effect of Atorvastatin is the substantial reduction in the level of atherogenic LDL3-7 subfractions. A shift towards larger, non-atherogenic LDL subfractions (LDL1 and LDL2) was also observed. Interestingly, similar effects on the transformation of the atherogenic phenotype were observed in overweight/obese women who were given apple/berry juice for six weeks [35]. The authors attributed this to a significant increase in plasma antioxidant status and magnesium levels. They hypothesised that apple/berry juice, rich in antioxidant phenols, may be beneficial as part of a healthy lifestyle, improving the lipid profile and thus contributing to a reduction in cardiovascular risk. In another study, Kalogirou et al. [36] observed a beneficial effect of combining Atorvastatin with ezetimibe on lipoprotein subfractions in patients with dyslipidaemia.

## 4. Materials and Methods

This was a prospective, observational, open-label study. Participation in this study had no effect on therapeutic decisions, invasive procedures, and the course of hospitalisation. This study received approval from the bioethical committee of the Medical University of Warsaw, Poland. All patients signed informed consent forms to participate in the research. This study was conducted during the autumn–winter season (from September to December) to avoid the effect of exposure to sunlight. The exclusion criteria were lack of written consent, not meeting the criteria for inclusion, paraneoplastic syndromes, severe anaemia (with a concentration of a blood haemoglobin less than 7 g/dL), history of chronic heart failure in New York Heart Association class III or IV, or the presence of acute severe heart failure (pulmonary oedema and/or cardiogenic shock).

### 4.1. Study Population

The study population consisted of 34 patients (10 females and 24 males; 55.97 ± 10.25 years) who had not previously been treated with lipid-lowering medications. Patients were undergoing the PCI procedure for the first time and had not previously experienced myocardial infarction. The study group included patients with first acute STEMI (n = 23) and NSTEMI (n = 11) who were admitted to the hospital within the first 3 h after the onset of symptoms. All patients underwent coronary angiography, followed by percutaneous coronary intervention (PCI) of the infarct-related coronary artery according to clinical indication. The demographic and clinical characteristics of patients after their first acute myocardial infarction are presented in Table 3. Atorvastatin (40 mg per day) was administered to all subjects as secondary prevention. Additionally, all patients were treated with aspirin and clopidogrel. Blood samples were collected from all patients twice during this study. The first whole-blood samples (n = 34) were taken after an overnight fast, the day after PCI, and before Atorvastatin treatment, and the second set (n = 34) was collected after 30 days ± 3 days of Atorvastatin therapy.

### 4.2. 25-(OH) Vitamin D Measurement

The concentration of 25(OH)D in serum samples was determined using direct competitive immunochemiluminescent assays and the DiaSorin Liaison^®^ automated system (DiaSorin Liaison, Sallugia, Italy). All samples were placed in the DiaSorin Liaison^®^ and run as directed by the manufacturer. During the incubation phase in the buffer, the 25(OH)D present in the sample dissociates from the binding protein and competes with vitamin D for binding sites on the anti-25(OH)D-coated particles. Incubation is followed by the measurement of the chemiluminescent signal.

### 4.3. Other Biochemical Assays (Lipid Profiles)

Serum lipid profiles (total cholesterol, TC; triglycerides, TG; and HDL cholesterol, HDL-Chol) were determined using a Cobas c501 automated analyser (Roche Diagnostics, Mannheim, Germany). The concentration of LDL cholesterol (LDL-Chol) was estimated using the Friedewald formula. Non-HDL cholesterol (Non-HDL-Chol) was calculated according to the appropriate formula [32]:

Non-HDL-Cholesterol = Total Cholesterol − HDL-Cholesterol

The lipid profiles obtained for each participant were then compared to the reference values provided by the 2024 Guidelines of the Polish Society of Laboratory Diagnostics and the Polish Lipid Association on laboratory diagnosis of lipid metabolism disorders [32]. Conversion of units from mg/dL to mmol/L was performed using the following conversion factors: (1) mg/dL × 0.02586 = mmol/L for total cholesterol, HDL, and LDL cholesterol; (2) mg/dL × 0.01129 = mmol/L for triglycerides [32].

### 4.4. Lipoprotein Subfractions

The Quantimetrix Lipoprint System LDL Subfractions Kit is a device designed to measure lipoprotein cholesterol (for lipoprotein fractions and subfractions from LDL to HDL) in fasting serum. The lipoprotein subfractions—VLDL, IDL-C, IDL-B, IDL-A, LDL1, LDL2 (large LDL), and LDL3-7 (small, dense LDL)—in the 34 patients were determined in serum using the Lipoprint^®^ analyser (Quantimetrix Corp., Redondo Beach, CA, USA) with the Quantimetrix Lipoprint System LDL Subfractions Kit (Quantimetrix, Redondo Beach, CA, USA) following the manufacturer’s instructions. This method uses linear electrophoresis on a non-denaturing polyacrylamide gel to separate and quantify lipoprotein subfractions. Based on the size of the LDL subfraction particles, the Lipoprint^®^ reports the LDL phenotype as non-atherogenic phenotype A (≥26.8 nm), intermediate phenotype AB (26.53–26.79 nm), and atherogenic phenotype B (≤26.5 nm). Individuals exhibiting lipoprotein profiles primarily consisting of larger, buoyant LDL1 and LDL2 subfractions are classified as Pattern A (phenotype A LDL, normal pattern LDL), while those with predominantly smaller, denser subfractions (LDL3–LDL7) are classified as Pattern B (phenotype B LDL, abnormal pattern LDL) [37].

### 4.5. Statistical Analysis

Continuous variables were tested for normal distribution using the Shapiro–Wilk test. Results for normally distributed continuous variables are expressed as mean ± standard deviation, and mean values were compared using an unpaired Student’s *t*-test. Differences were considered statistically significant at *p* < 0.05. Statistical analyses (verification of normal distribution, determination of mean and standard deviation and Spearman’s correlation coefficient, Rs) were performed using the statistical software STATISTICA version 13 and *p* values < 0.05 were considered statistically significant.

## 5. Conclusions

The presented data come from a non-randomised, open-label Atorvastatin study. Nevertheless, the results are interesting and suggest that the analysis of LDL subfractions provided a more detailed insight into lipid metabolism and enabled the identification of patients with a more atherogenic phenotype. LDL subfractions may thus become not only more precise and effective prognostic biomarkers, but also potential targets for lipid-lowering therapy. However, further studies are needed to identify the mechanisms involved and to determine the clinical usefulness of measuring LDL subfractions in the treatment of cardiovascular diseases, an area that remains controversial [38,39,40,41].

Furthermore, our data provide evidence that vitamin D deficiency is associated with atherogenic dyslipidaemia, particularly elevated levels of sdLDL (LDL3-7) levels, an important biomarker of cardiovascular risk. Another notable finding is that Atorvastatin is capable of inducing an increase in vitamin D biosynthesis.

## 6. Limitations

This study has two important limitations. First, the relatively small sample size necessitates further investigation with a larger cohort. Second, a treatment duration longer than one month of Atorvastatin therapy may be required. A clear limitation of our study is the absence of a control group of hyperlipidaemic subjects treated with Atorvastatin for primary prevention. Such a comparison would be helpful in identifying and understanding the effects of Atorvastatin not only on 25(OH)D levels but also on the clinical utility of lipoprotein subfraction analyses.

## Figures and Tables

**Table 1:** The effect of Atorvastatin treatment on conventional lipid profile.

	Pre-Atorvastatin Treatment	Post-Atorvastatin Treatment	*p* Value
Total Chol [mg/dL]	195.38 ± 38.36	132.15 ± 22.53	<0.0001
HDL-Chol [mg/dL]	54.97 ± 11.65	47.12 ± 13.47	0.0003
LDL-Chol [mg/dL]	116.24 ± 32.98	68.24 ± 14.98	<0.0001
Non-HDL-Chol [mg/dL]	140.41 ± 36.64	85.03 ± 18.36	<0.0001
Triglycerides [mg/dL]	139.41 ± 83.93	115.12 ± 62.36	0.1168

The results of lipid profile concentrations for all patients are presented as mean ± standard deviation.

**Table 2:** Triglyceride-rich lipoprotein (TRL) concentrations pre- and post-Atorvastatin treatment.

	Reference Range	Pre-Atorvastatin Treatment	Post-Atorvastatin Treatment	*p* Value
Triglycerides [mg/dL]	<100	139.41 ± 83.93	115.12 ± 62.36	0.1168
VLDL-Chol [mg/dL]	4.7–22.1	23.62 ± 7.12	16.82 ± 4.89	<0.0001
IDL-C Chol [mg/dL]	10.9–22.1	33.0 ± 8.74	22.2 ± 6.17	<0.0001
IDL-B Chol [mg/dL]	5.3–14.9	17.6 ± 6.43	11.1 ± 3.50	<0.0001
IDL-A Chol [mg/dL]	8.1–25.1	13.0 ± 4.91	7.50 ± 2.38	<0.0001

The results of triglyceride-rich lipoprotein concentrations for all patients are presented as mean ± standard deviation.

**Table 3:** Demographic and clinical characteristics of patients after the first acute myocardial infarction.

Characteristics	Patients (n = 34)
Age [years]	55.97 ± 10.25
Sex:	
Female, n (%)	10 (29.4%)
Male, n (%)	24 (70.6%)
Acute myocardial infarction	
STEMI, n (%)	23 (67.5%)
NSTEMI, n (%)	11 (32.4%)
Risk factors:	
Smoking, n (%)	17(50.0%)
Diabetes, n (%)	10 (29.4%)
Hypertension, n (%)	20 (58.8%)
Hypercholesterolaemia, n (%)	21 (61.7%)
Lipid profiles:	
Total cholesterol [mg/dL]	195.38 ±38.36
Triglycerides [mg/dL]	139.41 ± 83.93
25(OH)D [ng/mL]:	14.19 ± 6.37
25(OH)D ≥ 20 ng/mL, n (%)	6 (17%)
25(OH)D ˂ 20 ng/mL, n (%)	28 (83%)

Means ± standard deviations are summarised for continuous variables and frequencies and proportions are summarised for categorical variables. Abbreviations: STEMI—ST elevation myocardial infarction; NSTEMI—non-ST elevation myocardial infarction; 25(OH)D—25-hydroxy-vitamin D.

## Data Availability

The data presented in this study are available on request from the corresponding author.
